# Plateau Grass and Greenhouse Flower? Distinct Genetic Basis of Closely Related Toad Tadpoles Respectively Adapted to High Altitude and Karst Caves

**DOI:** 10.3390/genes11020123

**Published:** 2020-01-22

**Authors:** Liming Chang, Wei Zhu, Shengchao Shi, Meihua Zhang, Jianping Jiang, Cheng Li, Feng Xie, Bin Wang

**Affiliations:** 1 CAS Key Laboratory of Mountain Ecological Restoration and Bioresource Utilization & Ecological Restoration and Biodiversity Conservation Key Laboratory of Sichuan Province, Chengdu Institute of Biology, Chinese Academy of Sciences, Chengdu 610041, China; changliming16@mails.ucas.ac.cn (L.C.); zhuwei@cib.ac.cn (W.Z.); biomen@foxmail.com (S.S.); zhangmh@cib.ac.cn (M.Z.); licheng@cib.ac.cn (C.L.); xiefeng@cib.ac.cn (F.X.); 2 University of Chinese Academy of Sciences, Beijing 100049, China

**Keywords:** genetic adaptation, positively selected gene, fast evolving gene, high elevation, karst cave, environmental gradient

## Abstract

Genetic adaptation to extremes is a fascinating topic. Nevertheless, few studies have explored the genetic adaptation of closely related species respectively inhabiting distinct extremes. With deep transcriptome sequencing, we attempt to detect the genetic architectures of tadpoles of five closely related toad species adapted to the Tibetan Plateau, middle-altitude mountains and karst caves. Molecular evolution analyses indicated that not only the number of fast evolving genes (FEGs), but also the functioning coverage of FEGs, increased with elevation. Enrichment analyses correspondingly revealed that the highland species had most of the FEGs involved in high-elevation adaptation, for example, amino acid substitutions of XRCC6 in its binding domains might improve the capacity of DNA repair of the toad. Yet, few FEGs and positively selected genes (PSGs) involved in high-elevation adaptation were identified in the cave species, and none of which potentially contributed to cave adaptation. Accordingly, it is speculated that in the closely related toad tadpoles, genetic selection pressures increased with elevation, and cave adaptation was most likely derived from other factors (e.g., gene loss, pseudogenization or deletion), which could not be detected by our analyses. The findings supply a foundation for understanding the genetic adaptations of amphibians inhabiting extremes.

## 1. Introduction

The genetic adaptations of organisms inhabiting extreme environments have received increasing attention [1]. Extremes not only refer to the atypical levels of environmental factors, but also derive from the fluctuation degrees of these factors. It is suggested that species from more fluctuating environments show more rapid genetic evolution that might preadapt them to extremes [2,3]. Exploring how genetic evolution differs among extremes is undoubtedly interesting. The various extreme geological environments in China (e.g., Tibetan Plateau, Taklimakan desert and Southwest karst caves) harbors a mass of specialized species adapted to these extremes [4]. These conditions facilitate studies on the genetic backgrounds of lives adapted to distinct extreme environments.

The Tibetan Plateau, the largest high-elevation plateau, holds inhospitable environmental conditions such as low oxygen levels, high UV radiation levels, and low temperatures, as well as dramatic diurnal fluctuation of UV radiation and temperature. For adapting to these severe conditions, native dwellers often exhibit well-adapted characteristics, such as enhanced haemoglobin oxygen affinity, intensified pulmonary ventilation, and altered body size and skin structures [5,6,7]. Many studies have revealed the genetic architectures for high-elevation adaptation, with an emphasis on hypoxia adaption and energy metabolism in terrestrial animals, such as yak [8], Tibetan antelope [9], high-altitude birds [10,11], and highland frogs [12,13]. Nevertheless, a relatively small number of studies have focused on the genetic adaptation of aquatic animals living on the Tibetan Plateau [14,15].

The karst cave systems in Southwest China represent another kind of extreme environment characterized by permanent darkness, relative stable temperature and resource scarcity [16,17]. Cave animals often develop specialized morphological characteristics or behaviors, such as regression of eyes, reduction of pigmentation, high tactile sensitivity, and prolonged development duration [17,18]. Although a series of candidate genes (e.g., vision genes *Hsp 90α*, *Rh2-1, 2, 4,* and *Crystallin*) have been reported to be associated with cave adaptation in cavefishes [19,20,21,22], we still lack knowledge on the genetic adaptation in most other cave-dwellers.

Studies conducted to date have focused on the genetic adaptation to one of these two extreme environments (high elevation or cave systems), but not both, especially in the closely related species. Apparently, comparative analyses on the genetic evolution of one clade, whose habitats cover both the Tibetan Plateau and karst cave systems, are likely to give new insights into genetic adaptation. The Asian toad group of *Oreolalax* and *Scutiger* (Anura: Megophryidae) is an excellent model system. The two genera are phylogenetically sister groups [23], which have even been classified into one genus *Scutiger sensu lato* [24]. *Scutiger* toads mostly inhabit streams or wetlands at high elevations of ca. 3000–5000 m a.s.l. on the Tibetan Plateau and adjacent high mountains, while most species of *Oreolalax* are mainly distributed in the streams at middle or middle-to-high elevations of 1000–2800 m in the eastern edge of the Tibetan Plateau and Hengduan Mountains, in which only *O. rhodostigmatus* widely inhabits the karst cave systems at extensive elevations of 500–2200 m in Southwest China. *Scutiger* tadpoles can live in high-elevation waters for several years before metamorphosis. Meanwhile, *O. rhodostigmatus* tadpoles could survive several years in the darkness of the karst caves with a transparent body, though some individuals occasionally live in the outside mountain streams with a dark body, similar to tadpoles of other *Oreolalax* species [25,26]. The significant niche divergences would likely drive corresponding distinct genetic adaptation in the related toad species. Furthermore, to our knowledge, there is almost a gap in the genetic adaptation of tadpoles living in distinct extremes.

In this study, transcriptome sequencing is conducted for tadpoles of *O. rhodostigmatus* from karst cave environments, *O. omeimontis* and *O. popei* from mountain streams of 1300–1400 m, *O. major* from mountain streams of 2500 m, and *S. boulengeri* from Tibetan Plateau streams of 3700 m. Based on the data, we attempt to explore the genetic basis of high-elevation and cave adaptation in these related toad tadpoles. Moreover, we attempt to shed light on the different genetic basis of them, which is potentially associated with the degree of environmental fluctuations.

## 2. Materials and Methods

### 2.1. Ethics Statement

In this study, all experiments were performed according to the Guideline for the Care and Use of Laboratory Animals in China and proved by the Experimental Animal Use Ethics Committee of the Chengdu Institute of Biology.

### 2.2. Sampling

Tadpoles of *O. omeimontis*, *O. popei*, *O. major*, and *S. boulengeri* were collected from mountain streams at elevations of 1300, 1400, 2500 and 3700 m, respectively, from 10 May 2017 to 1 June 2017, while *O. rhodostigmatus* tadpoles were from a karst cave streams at an elevation of 1360 m on 15 May 2017, in the same breeding season (Table S1). For phylogenetic comparisons, *Megophrys omeimontis* tadpoles were collected from a mountain stream at 1200 m [27] (Table S1).

### 2.3. RNA Extraction and Sequencing

After being anesthetized by MS-222, tadpoles were sacrificed in order to collect liver, heart, skin and muscle tissues. For each species, tissues from three tadpoles were mixed as one sample for RNA extraction. Total RNA was extracted by TRIzol reagent (Invitrogen, Carlsbad, CA, USA) following the manufacturer’s protocol. RNA degradation and contamination were examined by electrophoresis. After purification, the concentration and integrity of RNA were quantified, and equal amounts of total RNA from the six species were used for constructing their respective cDNA libraries. Sequencing was performed on Illumina HiSeq 2500 platform. For comparisons, the whole set of DNA sequences of *Xenopus laevis* and *Nanorana parkeri* were obtained from the Ensembl database.

### 2.4. Quality Control, Sequence Assembly and Annotation

The reads quality was verified using FastQC v. 0.10.0 software [28]. Reads containing adapters, as well as poly-N and low-quality reads were removed. The total clean reads of each library were assembled de novo using Trinity as a reference transcriptome [29]. The sequencing data in this study have been submitted to the NCBI Sequence Read Archive (SRA) under accession number BioProject (PRJNA540463), and runs for each species including *M. omeimontis* (SRR8991302), *S. boulengeri* (SRR8991303), *O. popei* (SRR8991301), *O. rhodostigmatus* (SRR8991300), *O. major* (SRR8991299), and *O. omeimontis* (SRR8991298).

Blast2GO [30] software was used to obtain GO annotations defined by molecular function, cellular component and biological process ontologies. Pathway assignments were determined based on the KEGG database using KOBAS v. 3.0 [31,32] with an E-value threshold of 1.0 × 10^−5^. The CDS of each unigene was determined by either BLASTX against NR database or ESTSCAN [33]. After translation, the whole set of expressing protein sequences was obtained for *O. rhodostigmatus*, *O. major*, *S. boulengeri*, *M. omeimontis*, *O. omeimontis*, *O. popei*, *X. laevis*, and *N. parkeri*, respectively. The protein sequences of all these species were mixed as a sequence pool. Then, exhaustive pairwise BLASTP was conducted for this sequence pool, and the results were used to construct the orthologous sequence groups based on OrthoMCL v. 2.0.3 [34]. The single copy gene families (only one orthologs in each gene family) were extracted from the OrthoMCL database, and a total of 720 single copy gene families were identified across the eight species. For each gene family, their amino acid sequences were aligned and trimmed by MUSCLE [35] and Gblocks v. 0.91b [36] sequentially. These alignments were translated back into nucleotide sequences for further phylogenetic and evolutionary analyses.

### 2.5. Phylogenetic Reconstructions

Aligned sequences of all single copy orthologous genes were concatenated into a supergene using Python script. Phylogenetic trees were reconstructed using maximum likelihood (ML) and Bayesian inference (BI), as implemented in PHYML v. 3.0 [37] and BEAST v. 2.5.0 [38], respectively. *Xenopus laevis* and *N. parkeri* were used as outgroups. For ML and BI analyses, the GTR + I + G model was selected as the best substitution model by likelihood ratio tests under the Bayesian Information Criterion (BIC) implemented in JMODELTEST v. 2.1.7 [39]. In ML analyses, the default tree search approach using simultaneous Nearest Neighbor Interchange (NNI) method and BioNJ tree as the starting tree was used to estimate tree topologies. Non-parametric bootstrapping with heuristic searches of 10,000 replicates was used to assess confidences of branches. In BI analyses, two dependent runs were initiated each with four simultaneous Monte Carlo Markov chains (MCMC) for 50 million generations, with sampling every 1000 generations and discarding of the first 25% of generations as “burn-in”. Under a “relaxed molecular clock” assumption, divergence times between clades were estimated under an uncorrelated lognormal relaxed molecular clock model in BEAST. Calibration times obtained from the TimeTree project (www.timetree.org/) and/or previous studies [40] were used for divergence time estimates. We checked the convergence of the chains through visual inspection of plotted posterior estimates and by determining the effective sample size (ESS) for each parameter sampled from the MCMC analysis using the program Tracer v. 1.5 (tree.bio.ed.ac.uk/software/tracer/). The final tree, including divergence estimates and their 95% highest posterior densities (HPD), were computed in Treeannotator v. 1.4.5.

### 2.6. Accelerated Evolutionary Rates Analyses

The lineage-specific evolutionary rates for each branch were firstly estimated based on the genome-wide average values of dN, dS and dN/dS with the whole supergene dataset using the codeml program in PAML v. 4.9a [41]. On the other hand, 150 alignments were randomly selected and used for constructing supergenes and calculating the local dN, dS and dN/dS values. This process was replicated 10,000 times, and the resulting 10,000 local dN/dS values were used to present the distribution of the dN/dS ratios.

### 2.7. Identifying Fast Evolving Genes (FEGs) and Positively Selected Genes (PSGs)

FEGs were identified using the branch model in codeml program for each species. In this model, a null hypothesis assumes that all branches have the same evolutionary rate, and alternatively, the species in the foreground branch has a different evolving rate. The likelihood ratio test (LRT, df = 1) and Chisquare test were used to discriminate between alternative model for each orthologs in the gene set. Multiple testing was corrected by applying false discovery rate (FDR). Genes in the foreground branch having the higher dN/dS ratio and FDR-adjusted P value less than 0.05 were defined as candidate FEGs [14].

PSGs were also identified using branch-site model in codeml program for each species. The branch-site models aim to detect positive selection that affects only a few sites on prespecified lineages, which can be used to address two questions: the first is whether there are some sites in the gene that are under positive selection along the branches of interest, which is addressed by the LRT; and the second is to identify positively selected sites when they exist. This is achieved using the Bayes prediction [42]. By setting each of the branches as the foreground branch, the null hypothesis assumes sites on the foreground branch may evolve neutrally and under purifying selection. The alternative hypothesis assumes sites on the foreground branch may be under positive selection. LRT, Chisquare test and Bayes empirical Bayes (BEB) were used to discriminate between alternative models for each orthologs in the gene set. Genes with an FDR-adjusted P value less than 0.05 and posterior probability in excess of 0.95 were regarded as candidate PSGs [14].

Then, functional enrichment analyses (based on KOBAS) were carried out for FEGs or PSGs by querying against GO and KEGG databases.

### 2.8. Protein Structure Homology Modelling

We aligned the amino acid sequences of the candidate genes that were likely involved in adaptation to extremes obtained from RNAseq data of five toad species with the sequences from six model species from the Uniprot database including *Danio Retio*, *X. laevis*, *Gallus gallus*, *Mus musculus*, *Rattus norvegicus*, and *Homo sapiens* to observe amino acid substitutions in the toad species. Genes that had obvious mutations were chosen to build 3D structures of proteins. The 3D structure of proteins was constructed on Swiss-model server (www.swissmodel.expasy.org/) [43] and analysed on Swiss PDB Viewer.

## 3. Results

### 3.1. Gene Homology Analyses

**Figure 1:**
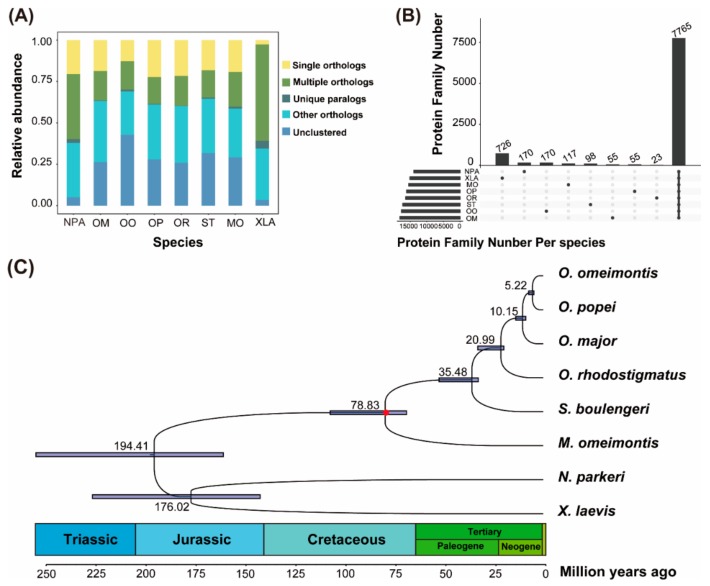
Orthologous genes and phylogenetic relationships between the eight amphibian species. (**A**) Relative abundance of different categories of orthologous gene families. Abbreviations: NPA, *Nanorana parkeri*; OM, *Oreolalax major*; OO, *O. omeimontis*; OP, *O. popei*; OR, *O. rhodostigmatus*; ST, *Scutiger boulengeri*; MO, *Megophrys omeimontis*; and XLA, *Xenopus laevis*. (**B**) Numbers of the common and unique gene families of the eight species. (**C**) Bayesian phylogenetic tree. The node bars indicate 95% posterior probability intervals of divergence time. The red dot denotes the calibration time point.

In total, 113,009, 130,280, 127,614, 104,456, 124,330, and 156,313 unigenes were obtained from transcriptomes of *O. rhodostigmatus*, *O. major*, *S. boulengeri*, *M. omeimontis*, *O. omeimontis*, and *O. popei*, respectively. The annotation rates were 34.14%, 30.70%, 34.86%, 38.26%, 44.09%, and 24.31%, respectively (Tables S2 and S3). Gene homology analyses grouped all unigenes into 27,356 orthologous gene families, of which 7765 gene families were shared by the eight species, including 720 putative single copy gene families (Table S4; Figure 1A,B; Figure S1).

### 3.2. Phylogenetic Reconstructions

Maximum likelihood (ML) and Bayesian inference (BI) analyses resulted in identical topologies, and all nodes were supported with almost full supports (all supports >99%; Figure 1 and Figure S1). *Scutiger boulengeri* was clustered as the sister of *Oreolalax* clade, and the divergence of them derived from about 35.48 mega-annus (Ma). In *Oreolalax*, *O. rhodostigmatus* was the sister to the clade containing the other three *Oreolalax* species with the relationship of (*O. major,* (*O. omeimontis, O. popei*)). The divergences of the four *Oreolalax* species derived from about 20.99 to 5.22 Ma.

### 3.3. Accelerated Evolutionary Rates Analyses

**Figure 2:**
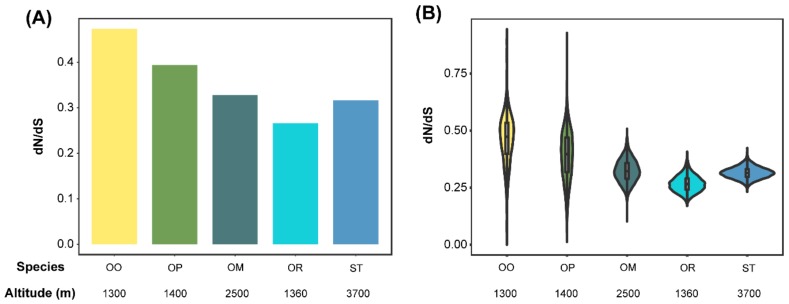
Comparisons of dN/dS ratios between toad species. (**A**) Global average dN/dS ratios (calculated by all single copy orthologous gene families). (**B**) Distribution of dN/dS ratios (calculated 150 randomly chosen orthologs, replicates = 10,000).

*Oreolalax omeimontis* and *O. rhodostigmatus* had the highest and lowest dN/dS ratios, respectively (*P* < 2.2 × 10^−16^, Wilcoxon rank sum test; Figure 2A). The distribution of dN/dS ratios showed the same pattern in *O. omeimontis* and *O. rhodostigmatus* (*P* < 2.2 × 10^−16^, Wilcoxon rank sum test; Figure 2B). In addition, the distribution of dN/dS ratios is dispersive in *O. omeimontis* and *O. popei,* but relatively concentrated in *S. boulengeri*, *O. major*, and *O. rhodostigmatus*.

### 3.4. Fast Evolving Genes (FEGs) and Positively Selected Genes (PSGs)

**Figure 3:**
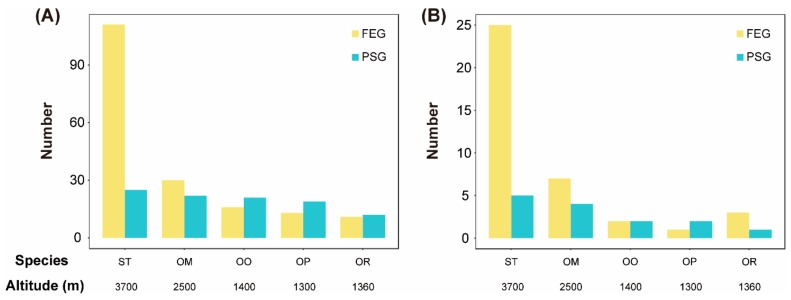
Comparisons of numbers of fast evolving genes (FEGs) and positively selected genes (PSGs) between toad species. (**A**) The total numbers of FEGs and PSGs. (**B**) The numbers of FEGs and PSGs functioning in mitochondria.

Molecular evolution analyses identified 111, 30, 16, 13, and 11 FEGs, as well as 25, 22, 21, 19, and 12 PSGs, from 720 single copy gene families in *S. boulengeri*, *O. major*, *O. omeimontis*, *O. popei*, and *O. rhodostigmatus*, respectively (Tables S5 and S6). Accordingly, the number of FEGs and PSGs tended to increase with the elevation (Figure 3A). This trend was also found when the focus was on FEGs and PSGs functioning in mitochondria (Figure 3B).

**Figure 4:**
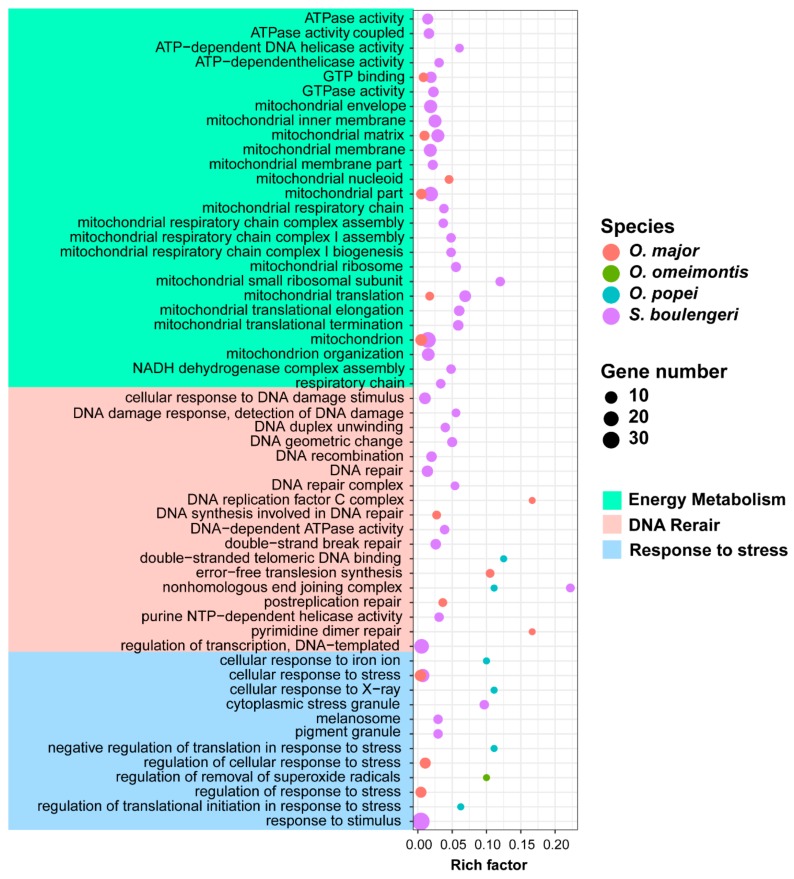
Gene Ontology (GO) terms enriched by FEGs related to high-elevation adaptation. The significant level is at 0.05. Rich factor is the ratio between number of genes enriched in a pathway and the total number of genes in this GO term.

The PSGs or FEGs of each toad species were queried against the gene ontology (GO) database (Tables S7 and S8). Rare GO terms related to high-elevation adaptation were significantly enriched by PSGs, while 42, 14, 6, and 1 ones were significantly enriched by FEGs in *S. boulengeri*, *O. major*, *O. omeimontis*, and *O. popei*, respectively, presenting a positive relationship between the numbers of these GO terms and the distribution attitudes of toad species. These GO terms could be clustered into three functional categories: “Response to stress”, “DNA repair”, and “Energy metabolism”, and the GO terms of high-elevation species, which tended to cover more functional categories (Figure 4).

### 3.5. Candidate Genes for Adapting to High Elevation and Karst Caves

Referring to existing literatures, a total of 40, 11, 8, 6 and 0 candidate genes were identified to be related to high-elevation adaptations in *S. boulengeri*, *O. major*, *O. omeimontis*, *O. popei,* and *O. rhodostigmatus*, respectively (Table 1). Unexpectedly, none of FEGs or PSGs were identified to be associated with cave adaptation in *O. rhodostigmatus*.

### 3.6. Amino Acid Substitutions of XRCC6 Gene in S. boulengeri

**Figure 5:**
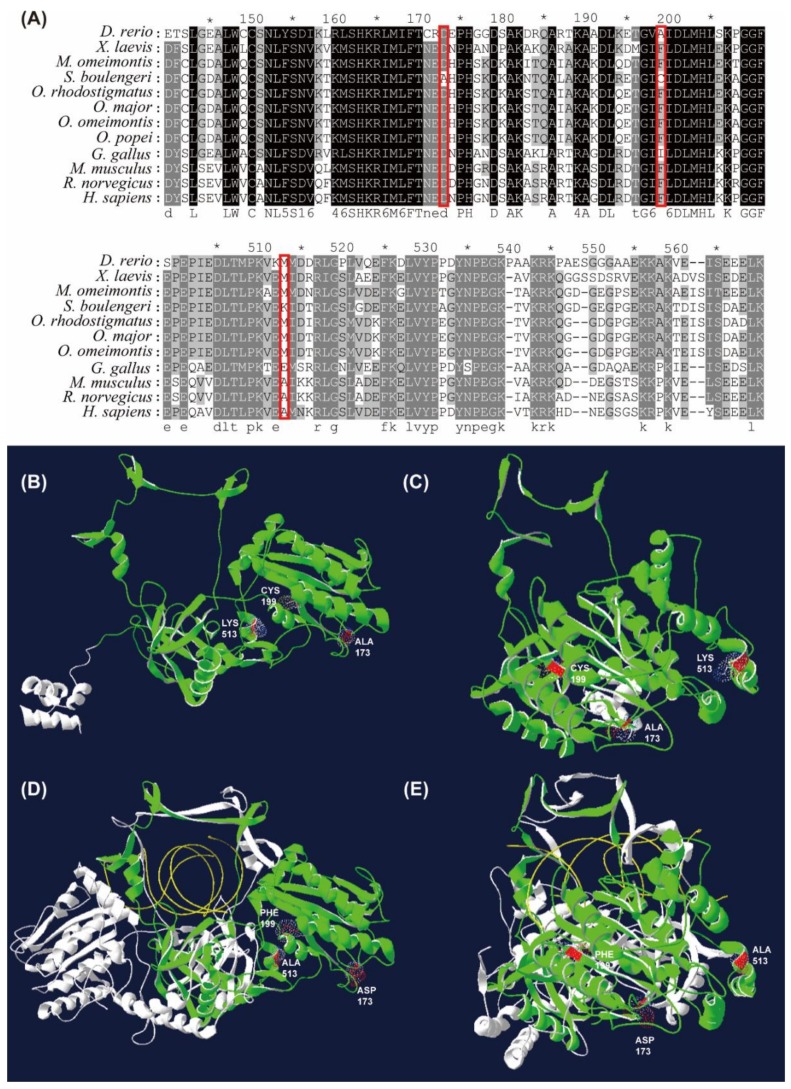
Amino acid substitutions of XRCC6 gene in *S. boulengeri* with respect to other vertebrates. (**A**) Alignments of XRCC6 amino acid sequences (numbered according to the *Homo sapiens* XRCC; red boxes indicate amino acid substitutions of XRCC6). (**B**,**C**) Vertical and lateral views of XRCC6 3D model in *S. boulengeri*. (**D**,**E**) Vertical and lateral views of 3D model in Human Ku dimer (Protein Data Bank accession numbers 1JEY). The substitution sites are denoted as red colour, the XRCC6 and XRCC5 subunits of Ku dimer as green and white colour, respectively, and the damaged DNA as yellow colour.

Remarkable amino acid substitutions (e.g., D173A, F199C, and M513K; numbered according to the *H. sapiens* XRCC; ID: jeq.1.A) were identified in the conserved domains of the X-ray repair cross-complementing protein 6 (XRCC6) in *S. boulengeri* (Figure 5A). Alignments showed that the D173A was located in a short loop domain, the F199C in a β-sheet domain, and the M513K in an α-helix domain (Figure 5B,C).

## 4. Discussion

Closely related species in one group inhabiting distinct extremes are expected to have a remarkably different genetic basis. Based on transcriptomics data, we detected the genetic architectures of tadpoles of five related representative species in the sister-genera *Oreolalax* and *Scutiger*, separately living in three kinds of environmental degrees: the high elevation of the Tibetan Plateau with dramatic environmental fluctuations, middle-elevation mountain streams with complicated environments in Hengduan Mountains, and karst cave systems with a relative stable environment in Southeast China. Some thought-provoking clues were brought from the results.

### 4.1. Genetic Signals for High-Elevation Adaptation of Toad Tadpoles on Tibetan Plateau

Tadpoles living at high elevations are exposed to low oxygen, high UV radiation, and low temperature. These environmental factors could have promoted the selection of genes involved in the “response to stimulus or stress”, “DNA repair”, and “energy metabolism.”

In coping with high levels of UV radiation on highlands, *S. boulengeri* was proposed to possess a well-developed pigment layer in both dorsal and ventral skin [44]. Our analyses identified FEGs involved in the “pigment granule” and “melanosome”, for example, the C10orf11 gene that encodes leucine-rich melanocyte differentiation-associated protein [45]. UV exposure can also damage DNA molecules by generating highly reactive chemical intermediates such as oxygen radicals [46]. On highlands, amphibians might be influenced by UV exposure more seriously than other animal categories due to their naked skin. In our study, many GO terms enriched by FEGs (i.e., RECQL, XRCC6, NSMCE1, RFC1, and DDX1) were related to the “DNA repair” in *S. boulengeri*. For instance, XRCC6 and XRCC5 constitute the Ku heterodimer, a critical component involved in the non-homologous end joining (NHEJ) pathway of DNA repair by binding with DNA double-strand break ends [47]. Our results suggested that three amino acid substitutions of XRCC6 in *S. boulengeri* were located in its binding domains with the XRCC5 (Figure 5D,E), which might improve its affinity with XRCC5 and the capacity of DNA repair in *S. boulengeri*.

Low oxygen is another typical limiting factor for the highland animals. Two candidate genes identified in *S. boulengeri* play important roles in hypoxia response. The first is the ATP-binding cassette sub-family B member 6 (ABCB6), which plays a key role in heme synthesis by binding with heme and porphyrins [48], and its fast evolution in *S. boulengeri*, which might improve the efficiency of heme synthesis, and thus promote the capacity of oxygen transportation. The other gene, CHCHD2, which is fast evolving in *S. boulengeri*, is a transcription factor that binds to the oxygen responsive element of COX4I2 and activates its transcription in response to hypoxia conditions [49].

Finally, low oxygen and low temperatures would likely lead to metabolic suppression, which challenge the capacity of highland animals to sustain core physiological functions [21,50]. Thus, it is necessary to improve the efficiency of energy production in mitochondrial to solve the contradiction between metabolic suppression and high energy supply. Mitochondria-related GO terms enriched by FEGs in *S. boulengeri* were involved in mitochondrial structure construction (e.g., mitochondria envelope, mitochondria membrane, mitochondria matrix, and mitochondria parts) and mitochondrial metabolic functions (e.g., mitochondria respiratory chain, NADH dehydrogenase complex assembly, ATPase activity, and GTPase activity) (Figure 4). The FEGs related to mitochondrial architecture included mitochondrial ribosomal proteins (e.g., MRPL37, MRPS35, MRPS7, MRPS28 and ERAL1) and translation factors (e.g., GFM1 and GFM2) [51,52], and the FEGs related to mitochondrial metabolic function included OXA1, C7orf55, NDUFA9, NDUFA12 and SLC25A10. Accelerated evolution of the former gene group might preadapt energy metabolism for the highlands from the aspect of the quantitative regulation of mitochondria biomass, while the latter from the aspect of qualitative catalytic dynamics.

### 4.2. Signals of High-Elevation Adaptation Increasing with Elevation in the Toad Species

Elevation divergence might drive genetic differentiation of animal species. For instance, Sun et al. (2018) detected that signals of genetic adaptation to high elevation increased from low-middle-elevation frogs and lizards to the high-elevation groups [12]. Our results also indicated this trend in the closely related toad tadpoles.

The number of FEGs and PSGs related to high-elevation adaptation increased with elevation, i.e., six genes, respectively, in *O. omeimontis* and *O. popei* occurring from 1300–1400 m, 11 in *O. major* from 2500 m, and sharply increasing to 42 in *S. boulengeri* from 3700 m. The increased gene number might reflect either broad selection pressures on more functional processes or concentrated selection on limited functional processes on highlands—our results seem to support the former. For *O. omeimontis* and *O. popei* from middle elevations, the identified FEGs and PSGs related to adaption to highlands were enriched in categories of “response to stress or stimulus”. In detail, five candidate genes were identified related to hypoxic response in *O. omeimontis* including PYROXD2, CRLS1, MTG1, GCH1 and EIF1AD, of which, PYROXD2, MTG1 and CRLS1 improve the rate of product energy, while GCH1 and EIF1AD might enhance the cellular response to oxidative stress [53,54,55]. In *O. popei*, candidate genes PYROXD1, TFR2 and TAMM41 were identified related to the “hypoxic response”. TFR2 encodes transferrin receptor 2, which mediates cellular uptake of transferrin-bound iron in a non-iron dependent manner and may be involved in iron metabolism, hepatocyte function and erythrocyte differentiation, which can improve the capacity to transport oxygen. XRCC6, a candidate gene relative to the “DNA repair”, plays an important role in UV response. For *O. major* tadpoles from 2500 m, four candidate genes HSF2, CHORDC1, DUSP22 and RAB8B, were identified that respond to acute environmental stressors. HSF2 encoded heat shock transcription factor 2 and CHORDC1 was proposed to act as co-chaperone for HSP90 and played a role in the regulation of NOD1 via a HSP90 chaperone complex [56]. DUSP22 activated the Jnk signaling pathway, and dephosphorylated and deactivated p38 and stress-activated protein kinase/c-Jun N-terminal kinase [57]. RAB8B is one of the members of RAS oncogene family. These genes help *O. major* to respond to dramatic environment fluctuations quickly. Three candidate genes associated with the “DNA Repair” (POLH, RFC1 and MRGBP) showed the UV response in *O. major*. Furthermore, four candidate genes involved in the “energy metabolism” (MTPAP, MTIF2, SCO1 and ERAL1), offered the higher energy production for *O. major*. The high-elevation species *S. boulengeri* obviously exhibited the most remarkable signs of possessing more genes for genetic adaptation to the Tibetan Plateau, as discussed above.

**Figure 6:**
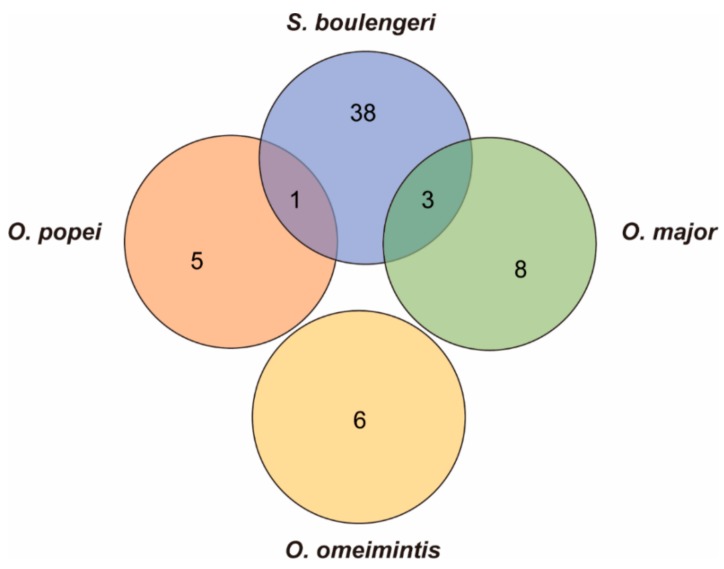
Venn diagram presenting the number of candidate genes associated with high-elevation adaptation in *S. boulengeri*, *O. major*, *O. popei*, and *O. omeimontis.*

In addition, only one gene (XRCC6) was identified both in the highland species *S. boulengeri* and the middle-elevation species *O. popei*, which was related to the “DNA repair”, and there were three candidate genes (MTIF2 and ERAL1 genes related to the “energy metabolism”, and RFC1 gene related to the “DNA repair”) recognized commonly in *S. boulengeri* and the middle-high-elevation species *O. major* (Figure 6). This also indicated the continued genetic evolution along the increasing elevations in the toads. Interestingly, XRCC6 was also screened as the candidate gene that is likely linked to adaptation to the high elevations in *Nanorana phrynoides* [12]. Furthermore, XRCC3 and XRCC4 are screened as the candidate genes that are likely linked to adaptation to the high elevations in *Phrynocephalus erythrurus* [58]. To summarize, XRCC protein plays an important role in high-elevation adaptation for amphibians and reptiles. It showed the convergent process on the pathway and divergent evolution on the molecules.

### 4.3. Explanations on Genetic Adaptation of the Cave Species O. Rhodostigmatus

*Oreolalax rhodostigmatus* tadpoles inhabit 500–2200 m mountains, and on elevations, broadly overlap with the tadpoles of *O. popei*, *O. omeimontis*, and *O. major*. However, the karst caves accommodate stable environments for *O. rhodostigmatus* tadpoles [26], keeping them away from the threats of UV radiation, diurnal and seasonal temperature fluctuations, and low oxygen above ground. Correspondingly, our analyses found no candidate gene involved in the “response to stimulus or stress” and “DNA repair” in *O. rhodostigmatus*, unlike that for the toad tadpoles above ground. In fact, we detected a few PSGs and FEGs in tadpoles of *O. rhodostigmatus*, but the small number of genes were only enriched in general GO terms, for example, “organelle part” and “intracellular organelle”.

Unexpectedly, our molecular evolution analyses also did not identify a candidate gene that potentially contributed to cave adaptation in *O. rhodostigmatus*. Thus, a series of typical phenotypes of cave adaptation in the toad tadpoles, such as small eyes, transparent bodies, and a long developmental cycle [17,18], might be derived from gene loss, pseudogenization or deletion [21], other than fast evolving or positive selection. For example, in *O. rhodostigmatus* tadpoles, in-frame deletion of four amino acids in the M/EJTD of MC1R supporting the transparent phenotype of the tadpoles [59], was not detected by our fast evolving and positively selective analyses. This indicated that further work on cave adaptation should focus on explorations of gene structure, multiple copy gene families, genomic architecture, etc.

## 5. Conclusions

Based on single-copy orthologous genes resulting from deep transcriptome sequencing, we explored the genetic profiles of tadpoles of five closely related toad species respectively adapted to the Tibetan Plateau, middle-elevation mountains, and karst cave systems. We found that the signals of genetic adaptation sharply increased with elevation and/or the degree of environmental fluctuations from cave species *O. rhodostigmatus*, middle-elevation species *O. omeimontis* and *O. popei*, middle-high-elevation species *O. major*, and highland species *S. boulengeri*. Of course, our analyses, especially the fast evolving and positively selected analyses, still could not rule out all genetic events adapting to the extremes, for instance, gene loss, pseudogenization, and deletions most likely existing in the cave tadpoles of *O. rhodostigmatus*. Future comparative analyses on whole genomes may promote explorations of the uncertainties. Overall, the findings provided a foundation for researching genetic adaptation of toads inhabiting distinct environments.

## Figures and Tables

**Table 1:** Candidate genes potentially associated with high-elevation adaptation.

Species	FEGs	PSGs
*S. boulengeri*	GFM1, MTIF2, ERAL1, GFM2, MRPL37, MRPS35, MRPS7, MRPS28, OXA1, C7orf55, NDUFA9, NDUFA12, SLC25A32, SLC25A10, RECQL, XRCC6, UBE2M, NSMCE1, RFC1, DDX1, CLDN3, SLC35C2, CHCHD2, ABCB6, CLPB, NPTX1, CHRND, SIGIRR, FAM58A, PMF1, AVEN, E2F6, C10orf11, VPS39	PDE12, ERAL1, NDUFA9, RECQL, AMN1, AVEN, CAPG, ARMC5, FAM58A, LRMDA, PSMB8, UBE2M, MRPS35
*O. major*	MTPAP, MTIF2, SCO1, ERAL1, POLH, RFC1, MRGBP, RAB8B, DUSP22	SCO1, ERAL1, RFC1, MRGBP, HSF2, DUSP22, IDS
*O. popei*	XRCC6, PYROXD1, TFR2, LACTB2, TECTA	ACP7, TAMM41, XRCC6, PYROXD1, TFR2, LACTB2, PINX1
*O. omeimontis*	PYROXD2, TSG101, EIF1AD, GCH1	MTG1, CRLS1, GCH1

## Supplementary Materials

The following are available online at https://www.mdpi.com/2073-4425/11/2/123/s1, Table S1: Information of samples used in this study, Table S2: Unigene annotation details, Table S3: Summary of sequencing quality, Table S4: Summary of orthologous groups among eight species, Table S5: Fast evolving genes (FEGs) list, Table S6: Positive selection genes (PSGs) list, Table S7: Summary of GO enrichment based on stricter FEGs, Table S8: Summary of GO enrichment based on stricter PSGs, Figure S1: The phylogenetic relationship of species.
