# Supplementary figures and images for: Plateau Grass and Greenhouse Flower? Distinct Genetic Basis of Closely Related Toad Tadpoles Respectively Adapted to High Altitude and Karst Caves
Source: Genes (Basel). 2020 Jan 22;11(2):123. doi: 10.3390/genes11020123 (PMC7073644; doi:10.3390/genes11020123)

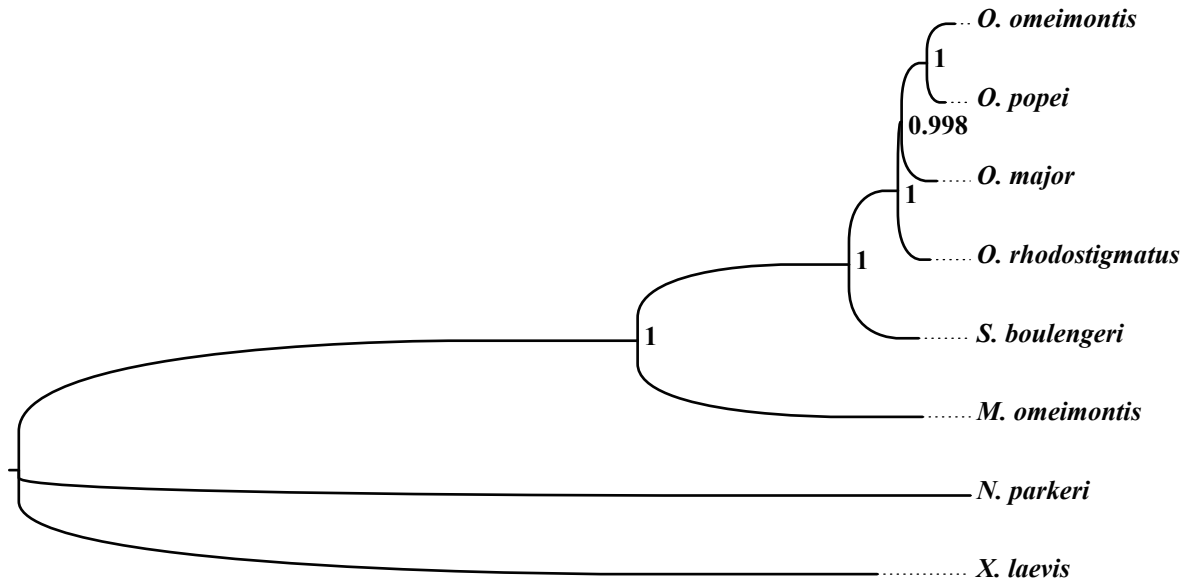

Supplement: Supplementary file 1 [file genes-11-00123-s001.zip › genes-670519-supplementary/Figure S1. The phylogenetic relationship of species..pdf]
